# The Impact of Multidisciplinary Team Intervention for Early Mobilization of Patients with Aneurysmal Subarachnoid Hemorrhage in Stroke Care Unit: A Retrospective Cohort Study

**DOI:** 10.1298/ptr.E10297

**Published:** 2024-11-28

**Authors:** Kenji OIKE, Osamu ISHIBASHI, Nobuyuki NOSAKA, Shin HIROTA

**Affiliations:** 1 Department of Rehabilitation, Tsuchiura Kyodo General Hospital, Japan; 2 Department of Intensive Care Medicine, Tokyo Medical and Dental University, Japan; 3 Department of Neurosurgery, Tsuchiura Kyodo General Hospital, Japan

**Keywords:** Aneurysmal subarachnoid hemorrhage, Early mobilization, Multidisciplinary team intervention, Stroke care unit

## Abstract

Objective: To investigate the impact of multidisciplinary team (MDT) intervention for early mobilization (EM) of patients with aneurysmal subarachnoid hemorrhage (aSAH) in the intensive care unit (ICU). Methods: A retrospective uncontrolled before–after observational study was conducted to assess patient outcomes before and after introducing MDT in the stroke care unit (SCU). Participants admitted to the SCU from April 2017 to September 2023 were categorized into conventional (April 2017 to June 2020) and MDT (July 2020 to September 2023) groups. The measured primary outcome was the days until sitting, standing, and walking commenced. Results: A total of 131 patients were screened, with 115 included in the analysis. The MDT group comprised 56 individuals (48.7%), whereas the conventional group consisted of 59 patients (51.3%). The MDT group exhibited a significantly shorter duration until sitting (4 [3–7] vs. 7 [5–17], p <0.001), standing (5 [3–7] vs. 10 [5–17], p <0.001), and walking (7 [5–10] vs. 16 [7–23], p <0.001) commenced. Furthermore, the MDT group showed a significantly higher ICU mobility scale (IMS) (8 [5–8] vs. 5 [3–8], p <0.001) at SCU discharge, shorter length of SCU stay (16 [15–17] vs. 17 [15–24], p = 0.048), and hospital stay (34 [25–48] vs. 48 [33–80], p = 0.006). Conclusion: This study suggests that MDT played a facilitative role in promoting the EM of patients with aSAH. Their involvement streamlined the mobilization process, shortening the days until the initiation of mobilization.

## Introduction

Aneurysmal subarachnoid hemorrhage (aSAH) accounts for 5% of all strokes^1)^; however it can affect even the young population and has a high mortality rate as high as 30%^2^^,^^3)^. The long-term impact is also significant, with nearly half of the patients facing functional disability at hospital discharge^4)^. Accordingly, strict intensive care management is crucial after an aneurysm repair to prevent unique complications, such as symptomatic cerebral vasospasm, rebleeding, and hydrocephalus^5)^.

Previous guidelines did not recommend early mobilization (EM) for aSAH^6^^,^^7)^. Due to symptomatic vasospasm developing within 14 days of onset, EM is not aggressively implemented in daily intensive care^8)^. Cerebral vasospasm was reported to result in delayed cerebral ischemia 2–5 days aSAH onset^9)^, complicating the EM standardization for aSAH in Japan. However, observational studies have suggested that EM is feasible without adverse events^4^^,^^10^^–^^14)^, and recent guidelines have mentioned the benefits of EM^15)^.

The difficulty in standardizing EM forces patients to remain on bed rest for long periods. Prolonged immobility is associated with reduced neuromuscular function^16)^ as well as increased cardiopulmonary dysfunction^17)^. Additionally, aSAH patients are susceptible to muscle mass decrease^18)^ and secondary complications related to immobility such as pneumonia^19)^ and deep venous thrombosis (DVT^20)^), leading to declines in cognitive and physical function^21)^. Accordingly, immobility-related complications may make recovery from SAH even more difficult in addition to disease-specific complications.

In this challenging situation, multidisciplinary team (MDT) interventions may play a crucial role^8)^. MDT intervention for aSAH might reduce the length of hospital stay^22)^ and facilitate appropriate discharge^23)^. However, reports on the MDT intervention for patients with aSAH in the ICU are limited. Our hospital provides acute postoperative care for patients with aSAH in a unit known as the stroke care unit (SCU), equivalent to the stroke unit defined by the European Stroke Organization^24)^. All staff in the SCU are neurological experts.

Given these circumstances, we aimed to investigate the impact of MDT intervention on postoperative mobilization for aSAH in the SCU and assess clinical outcomes at hospital discharge.

## Methods

### Setting

Tsuchiura Kyodo General Hospital, licensed for 800 inpatient beds, includes an SCU with 8 registered beds, which is the focus of this study. The SCU is staffed with in-house physicians available 24/7, and registered nurses maintain a nurse-to-patient ratio not exceeding 1:4.

All aSAH patients were admitted to the SCU for the first 14 days of cerebral vasospasm without exception. They were discharged once their general condition stabilized, particularly concerning respiratory status and severe delirium.

### PT intervention

Before July 2020, patients in the SCU received a 20-min rehabilitation intervention daily, under individual instruction by physicians, determined by the physical therapists (PTs) in charge. Post-SCU discharge, patients underwent basic movement and gait exercises once or twice a day for 20–40 min.

### MDT intervention for EM

In July 2020, PTs were introduced to the SCU (SCU PTs), forming an MDT alongside physicians, nurses, and clinical engineers. The roles of each profession on the MDT include the following^25)^: physicians who determine suitability for EM, SCU PTs who plan and implement EM protocols to improve physical function, nurses who coordinate multidisciplinary collaboration and consider safety during EM interventions, and clinical engineers who ensure the safety of machines such as ventilators.

**Fig. 1.**
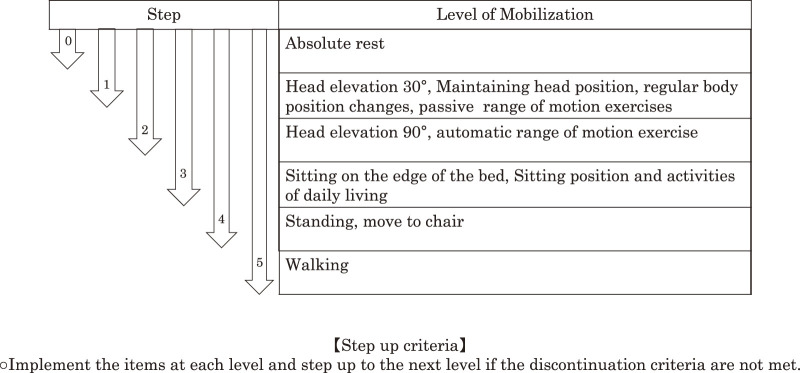
Early mobilization protocol The items at each level are implemented and step up to the next level if the discontinuation criteria are not met

In accordance with the initiation of MDT, an EM protocol was developed, referencing previous reports^25^^,^^26)^ (Fig. 1). The protocol involves the EM team creating a mobilization plan for each patient upon SCU admission, with an initial assessment within 48 h. EM initiation began at level 2 unless the patient met the non-intervention criteria (Table 1), with step-up criteria implemented daily if discontinuation criteria were not met (Table 2). Patients in the SCU received a 20–30 min EM intervention daily by MDT.

**Table 1.** Criteria for not starting early mobilization

PaO_2_/FiO_2_	Less than 250
PEEP (cmH_2_O)	10 or more
SpO_2_ (%)	90 or less
RR (times/min)	Other than 10–30
Initial myocardial ischemia	
HR (bpm)	Other than 50–120
MAP (mmHg)	Other than 55–140
SBP (mmHg)	Other than 90–180
Vasopressor injection	New or increased within 24 h
RASS	–3 or less
Instruction to rest with a head elevation of less than 30° or prohibition of changing body position

PaO_2_, partial pressure of arterial oxygen; FiO_2_, fraction of inspiratory oxygen; PEEP, positive end expiratory pressure; SpO_2_, oxygen saturation of peripheral artery; RR, respiratory rate; HR, heart rate; MAP, mean arterial pressure; SBP, systolic blood pressure; RASS, Richmond Agitation Sedation Scale

**Table 2.** Discontinuation criteria

1) Respiratory status (index)
Respiration rate <5 times/min, >40 times/min
SpO_2_ <90%
2) Circulatory dynamics (indicators)
HR <40 beats/min, >140 beats/min Separate evaluation for atrial fibrillation
Emergence of emergency
Hypertensive blood pressure >180 mmHg
MAP <65 mmHg, >110 mmHg
3) Consciousness symptoms (indicators)
Increase the amount of sedatives
Feeling of difficulty breathing during labor
Appearance of chest symptoms
Patient refusal

SpO_2_, oxygen saturation of peripheral artery; HR, heart rate; MAP, mean arterial pressure

EM rounds, conducted twice daily, ensured proper protocol operation and mobilization intervention by SCU PTs and nurses.

### Before-after analysis of patient outcomes

**Fig. 2.**
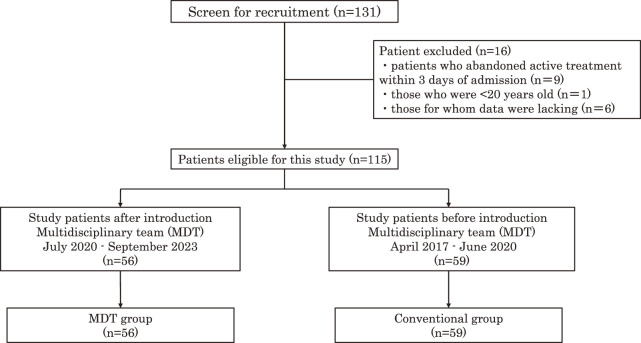
Flow chart of study participation A total of 131 participants were included in the initial patient pool. After applying the exclusion criteria, 115 participants were included in the analysis

A retrospective uncontrolled before-after observational study assessed patient outcomes before and after the introduction of MDT in the SCU. Participants admitted to the SCU from April 2017 to September 2023 were divided into the conventional (April 2017 to June 2020) and MDT (July 2020 to September 2023) groups (see Fig. 2). Collected data included patient demographics, World Federation of Neurosurgical Societies (WFNS) grades^27)^, aneurysm location, rehabilitation duration, sitting, standing, and walking days, ICU mobility scale (IMS)^28)^ at SCU discharge, neurological complications, length of hospital stay, modified Rankin scale^29)^ at discharge, and discharge disposition.

### Statistical analysis

Continuous variables were subjected to the Shapiro–Wilk test for normality, expressed as means ± standard deviations for normally distributed indicators and median (interquartile range) for non-normally distributed indicators. Categorical variables were presented as frequencies (%). Two-group comparisons used unpaired t-tests or Mann–Whitney U tests based on normality, and the chi-square test for categorical variables. Statistical analysis used EZR (ver. 1.52)^30)^, setting significance at <5%.

### Ethics statement

The study obtained approval from the ethics committee of Tsuchiura Kyodo General Hospital (2023FY17) and adhered to the Declaration of Helsinki principles and Strengthening the Reporting of Observational Studies in Epidemiology guidelines. Participants received information about the research (opt-out), ensuring the opportunity to refuse participation.

## Results

**Fig. 3.**
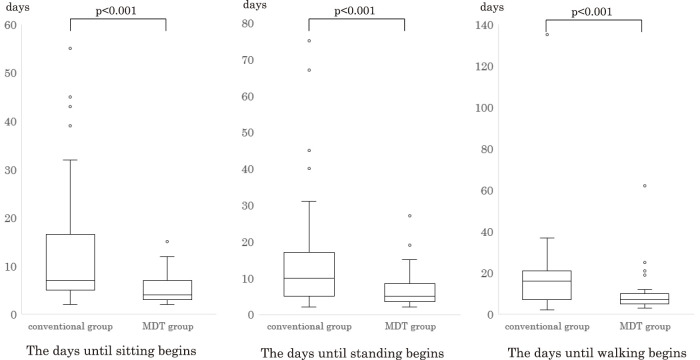
Days until sitting, standing, and walking begin The MDT group exhibited a significant reduction in the days until sitting, standing, and walking. MDT, multidisciplinary team

**Fig. 4.**
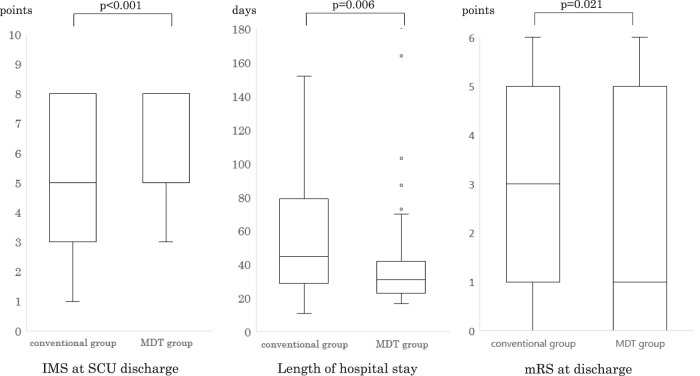
The ICU mobility scale at SCU discharge, length of SCU stay, and modified Rankin scale at hospital discharge The MDT group showed a significantly higher IMS at SCU discharge, shorter SCU stay, and length of hospital stay. MDT, multidisciplinary team; ICU, intensive care unit; IMS, ICU mobility scale; SCU, stroke care unit; mRS, modified Rankin scale

A total of 131 patients underwent screening, with 115 included in the analysis. Figure 2 shows the flowchart of patient selection. The MDT group comprised 56 individuals (48.7%), while the conventional group consisted of 59 patients (51.3%). Patient backgrounds and EM progress in the SCU are presented in Table 3, Figures 3 and 4, respectively. The MDT group exhibited a significantly shorter duration until rehabilitation initiation (2 [2–3] vs. 3 [2–4], p <0.001). Additionally, the MDT group demonstrated significant reductions in the days until sitting (4 [3–7] vs. 7 [5–17], p <0.001), standing (5 [3–7] vs. 10 [5–17], p <0.001), and walking (7 [5–10] vs. 16 [7–23], p <0.001) commenced. Moreover, the MDT group showed a significantly higher IMS (8 [5–8] vs. 5 [3–8], p <0.001) at SCU discharge. Interestingly, the MDT group experienced a significantly shorter length of SCU stay (16 [15–17] vs. 17 [15–24], p = 0.048) and length of hospital stay (34 [25–48] vs. 48 [33–80], p = 0.006). No significant differences in neurological complications, patient backgrounds, or discharge disposition were observed between the 2 groups.

**Table 3.** Baseline characteristics and comparison between study groups

	Total (n = 115)	MDT group (n = 56)	Conventional group (n = 59)	p-Value
Age (years)	64.9 ± 15.4	64.1 ± 15.0	65.7 ± 15.9	0.572^††^
Male, n (%)	37 (32.2%)	22 (39.3%)	15 (25.4%)	0.125^†††^
WFNS grade				
I–III	69 (60.0%)	35 (62.5%)	34 (57.6%)	
IV–V	46 (40.0%)	21 (37.5%)	25 (42.4%)	0.656^†††^
Aneurysm location, n (%)				
Anterior location	95 (82.6%)	44 (78.6%)	51 (86.4%)	
Posterior location	20 (17.4%)	12 (21.4%)	8 (13.6%)	0.283^†††^
Type of aneurysmal treatment, n (%)				
Surgical clip ligation	53 (46.1%)	30 (53.6%)	23 (39.0%)	
Endovascular coil embolization	62 (53.9%)	26 (46.4%)	36 (61.0%)	0.358^†††^
Duration of mechanical ventilation, days	2 (1–4)	2 (1–4)	2 (1–4)	0.630†
Complications, n (%)				
Symptomatic cerebral vasospasm	15 (13.0%)	7 (12.5%)	8 (13.6%)	0.838^†††^
Rebleeding	7 (6.3%)	3 (5.4%)	4 (6.8%)	0.732^†††^
Hydrocephalus	33 (31.3%)	14 (25.0%)	22 (37.3%)	0.137^†††^
The number of days until rehabilitation begins	3 (2–4)	2 (2–3)	3 (2–4)	<0.001^†**^
Length of SCU stay, days	16.0 (15.0–19.0)	16.0 (15.0–17.0)	17.0 (15.0–23.8)	0.048^†*^
Discharge to home (%)	51 (44.3%)	27 (48.2%)	24 (40.6%)	0.463^†††^

Mean ± standard deviation, median (interquartile range).

†Mann–Whitney U test; ††Student's t-test; †††Chi-squared test; *p <0.05; **p <0.001.

MDT, multidisciplinary team; WFNS, World Federation of Neurosurgical Societies; SCU, stroke care unit

## Discussion

This observational study, conducted in a single tertiary care hospital in Japan, illustrated the effectiveness of MDT in the SCU on postoperative mobilization among postoperative patients with aSAH. Notably, in the MDT group, EM was promoted to enhance physical function upon the patient’s discharge from the SCU, shorten the length of hospital stay, and improve ADLs at the time of the patient’s hospital discharge. Although previous studies have reported that EM for patients with aSAH is associated with better outcomes, the means to promote EM were unclear. This study is of clinical significance as it revealed that MDT intervention based on the established EM protocol in the SCU facilitated EM for aSAH.

In intensive care, EM obstacles have been documented, including deficient communication among relevant professionals and a lack of coordination during rounds^31)^. Intriguingly, however, this study showed that the MDT group implemented protocols and conducted collaborative rounds involving various professions, fostering a collective understanding of the intervention and facilitating EM. The physicians gave comprehensive instructions for the EM, and the SCU PTs played a central role in the EM, acting as facilitators. The nurses were responsible for adjusting the schedule and managing safety for EM, and the clinical engineers were responsible for adjusting devices, including ventilator management, which allowed for safe and effective EM. This study revealed that integrating an EM protocol with MDT enabled multidisciplinary collaboration, promoting timely mobilization. Effective team-based care necessitates a facilitator within the system^32)^. Alongside system implementation, the presence of a facilitator is crucial for smooth operation. This streamlined the mobilization process, ultimately reducing the time until mobilization initiation.

The study results demonstrated that EM for aSAH effectively improves patient outcomes. In addition, our patients did not develop symptomatic cerebral vasospasm, which has been suggested to occur within 14 days postoperatively, indicating that EM can be safely performed and that our experience reinforces previous reports^4^^,^^10^^–^^14^^,^^33)^.

This study has certain limitations. First, as it is a single-center case–control study, its generalizability is restricted. Second, the scope of mobilization intervention is confined to the SCU. That is, the EM intervention after discharge from the SCU was not standardized. This might contribute to the result of the unchanged rate of returning home between groups. In addition, data regarding the status of family support or the structure of the home were unavailable in this study. Factors related to returning home have been reported^34)^ to include physical function as well as family and social circumstances. Accordingly, future studies should aim to comprehensively assess mobilization interventions by evaluating the overall hospitalization period and considering the patients’ surrounding living conditions.

This evaluation is crucial for understanding their potential effects on ambulation ability at discharge.

## Conclusion

This study found that MDT intervention in the SCU may contribute to earlier mobilization of patients with aSAH than those without MDT. Furthermore, the EM may have enhanced physical activity at SCU discharge, potentially translating into improved ADLs at discharge and a shorter hospital stay.

## Acknowledgments

The authors thank Naomi Shibasaki, Emiko Kayaba, and Ippei Hamano at Tsuchiura Kyodo General Hospital for their assistance with this study.

## Funding

Not applicable.

## Conflicts of Interest

None.
